# Variability of trunk muscle synergies underlying the multidirectional movements and stability trunk motor tasks in healthy individuals

**DOI:** 10.1038/s41598-023-28467-6

**Published:** 2023-01-21

**Authors:** Hiroki Saito, Hikaru Yokoyama, Atsushi Sasaki, Kazuya Matsushita, Kimitaka Nakazawa

**Affiliations:** 1 Department of Life Sciences, Graduate School of Arts and Sciences, The University of Tokyo, Tokyo, Japan; 2 Department of Physical Therapy, Tokyo University of Technology, Tokyo, Japan; 3 Institute of Engineering, Tokyo University of Agriculture and Technology, Tokyo, Japan; 4 Department of Mechanical Science and Bioengineering, Graduate School of Engineering Science, Osaka University, Osaka, Japan; 5 Aobadai Takeda Orthopedic, Tokyo, Japan; 6 Japan Society for the Promotion of Science, Tokyo, Japan

**Keywords:** Motor control, Neurophysiology

## Abstract

Muscle synergy analysis is useful for investigating trunk coordination patterns based on the assumption that the central nervous system reduces the dimensionality of muscle activation to simplify movement. This study aimed to quantify the variability in trunk muscle synergy during various trunk motor tasks in healthy participants to provide reference data for evaluating trunk control strategies in patients and athletes. Sixteen healthy individuals performed 11 trunk movement and stability tasks with electromyography (EMG) recording of their spinal and abdominal muscles (6 bilaterally). Non-negative matrix factorization applied to the concatenated EMG of all tasks identified the five trunk muscle synergies (W) with their corresponding temporal patterns (C). The medians of within-cluster similarity defined by scalar products in W and r_max_ coefficient using the cross-correlation function in C were 0.73–0.86 and 0.64–0.75, respectively, while the inter-session similarities were 0.81–0.96 and 0.74–0.84, respectively. However, the lowest and highest values of both similarity indices were broad, reflecting the musculoskeletal system’s redundancy within and between participants. Furthermore, the significant differences in the degree of variability between the trunk synergies may represent the different neural features of synergy organization and strategies to overcome the various mechanical demands of a motor task.

## Introduction

Trunk control plays an essential role in human movement. Optimal control during daily activities, such as locomotion and highly skilled sports performance, requires stability and movement of the trunk in response to a specific demand^1^. Therefore, the spinal and abdominal muscles with different roles should be well-coordinated to address redundancy in the musculoskeletal system^2^. This perspective is based on the premise that the central nervous system (CNS) relies on a limited number of motor modules or muscle synergies formed by flexible combination of muscle activation as the most effective way to select the control signal from a large subspace^3–5^.

Dimensionality reduction algorithms, such as non-negative matrix factorization (NMF), are useful for extracting embedded structures as muscle synergies from the variability of electromyography (EMG)^6–9^. We have previously identified five basic units of trunk muscle synergies from 12 trunk muscles (i.e., six muscles bilaterally) during 24 locomotion and stability tasks in the trunk and lower limbs^10^. In this study, it was hypothesized that the CNS flexibly selects and modifies the degree of contribution to a few sets of basic trunk muscle synergies rather than framing a new pattern to overcome several human locomotion and stability motor behaviors^10^.

We consider the analysis of the basic units of trunk muscle synergies useful for improving our understanding of trunk motor control strategies in a target population, including individuals with neurological and musculoskeletal conditions, and athletes^10^. The study design considered the various functional postures of the trunk and physiological variability within tasks, and it minimizes the likelihood of biomechanical constraints (e.g., muscle moment arms and skeletal geometry) leading to consistent activation of different groups of muscles, which results in multiple synergies inadequately merged into a single synergy^11,12^. Although the extent to which EMG variability is necessary for the accurate estimation of synergy has remained undefined^13^, NMF for such large-scale and high-dimensional EMG data can help to infer specific muscle synergies to represent neural constraint on movement rather than those reflecting biomechanical constraints^12,13^. Thus, in the clinical field, this method addresses the question of how the CNS adapts to motor control under several conditions, such as diseases in patients or motor training in athletes. Furthermore, our previous study included 11 functional tasks of multidirectional trunk movements and several of the four positional tasks that require trunk stability^10^. Each motor task has largely been utilized in research and clinical fields to evaluate and improve motor control^14–18^. Therefore, the cluster of trunk motor tasks increases the likelihood of revealing the motor control strategies in a target population that may have diverse features of altered trunk motor control^14^ and help to interpret several meaningful daily and sports activities^10^.

For clinical application, assessments using trunk muscle synergies require the extraction of synergies from data under different conditions (e.g., patients vs. healthy participants) during a single session or multiple sessions for longitudinal studies, which compare synergies to investigate different control strategies or track changes in motor performance and recovery^19–21^. To effectively identify the trunk muscle synergy patterns in a target population, we first need to quantify the variability in trunk synergy in healthy participants, especially when exploring a large subset of movement^19,22^. In this context, variability is defined as the range of variations in trunk muscle synergies between participants during motor tasks within a session (inter-participant variability) and it reflects the likelihood of each individual achieving motor outputs with different recruitment muscle patterns^23,24^. Thus, it helps to assess the stereotypes or diverse features of trunk motor control strategies in the population of interest and interpret the adaptability in the presence of diseases, and understand the process of motor training^25–29^. Other aspects of variability focused on the variations within a participant between sessions (inter-session variability) in which a particular individual shows natural session-by-session variability of motor performances^30^. Inter-session variability is crucial as it provides reliable assessments to interpret whether changes in muscle synergies after interventions are considered as natural, subjective variability, or to improve motor functions^19^.

It is also important to investigate the difference in the degree of variability between synergies to understand the characteristics of muscle synergy organization. It was found that muscle pairs that are anatomically and functionally closely related increase their connectivity when they share origin, insertion, and nerve supply^31^. This may suggest that muscle synergies are strongly embedded structures with low-level neural control, and the variability of muscle synergy organization is expected to be low^11,32^. In contrast, if the variability of muscle synergy organization is high, it suggests that the synergies are flexibly modulated by the upstream layer of supraspinal circuits, which may reflect the sensorimotor experience of the individual during development^13,33^. Furthermore, a previous study found that the degree of variability differed depending on the synergies activated in different planes of movement of the upper limbs^22^. Trunk muscle synergies have different activation patterns depending on the 11 motor tasks that require various features of movement and postural stability. Therefore, investigating the difference in the degree of variability between synergies will help interpret the functional roles of each muscle synergy to overcome the specific demands of each task^26,27^.

Several studies have investigated the variability of muscle synergies for different scenarios, such as locomotion^34,35^, upper arm reaching^22^ and daily activities^20^ to provide reference data sets for clinical utilization. However, the variability of trunk synergies underlying diverse trunk motor behaviors has not yet been analyzed. Therefore, the purpose of the current study was to investigate the variability in trunk muscle synergies of healthy participants during 11 trunk movements and stability tasks.

## Methods

### Experimental protocol

**Figure 1:**
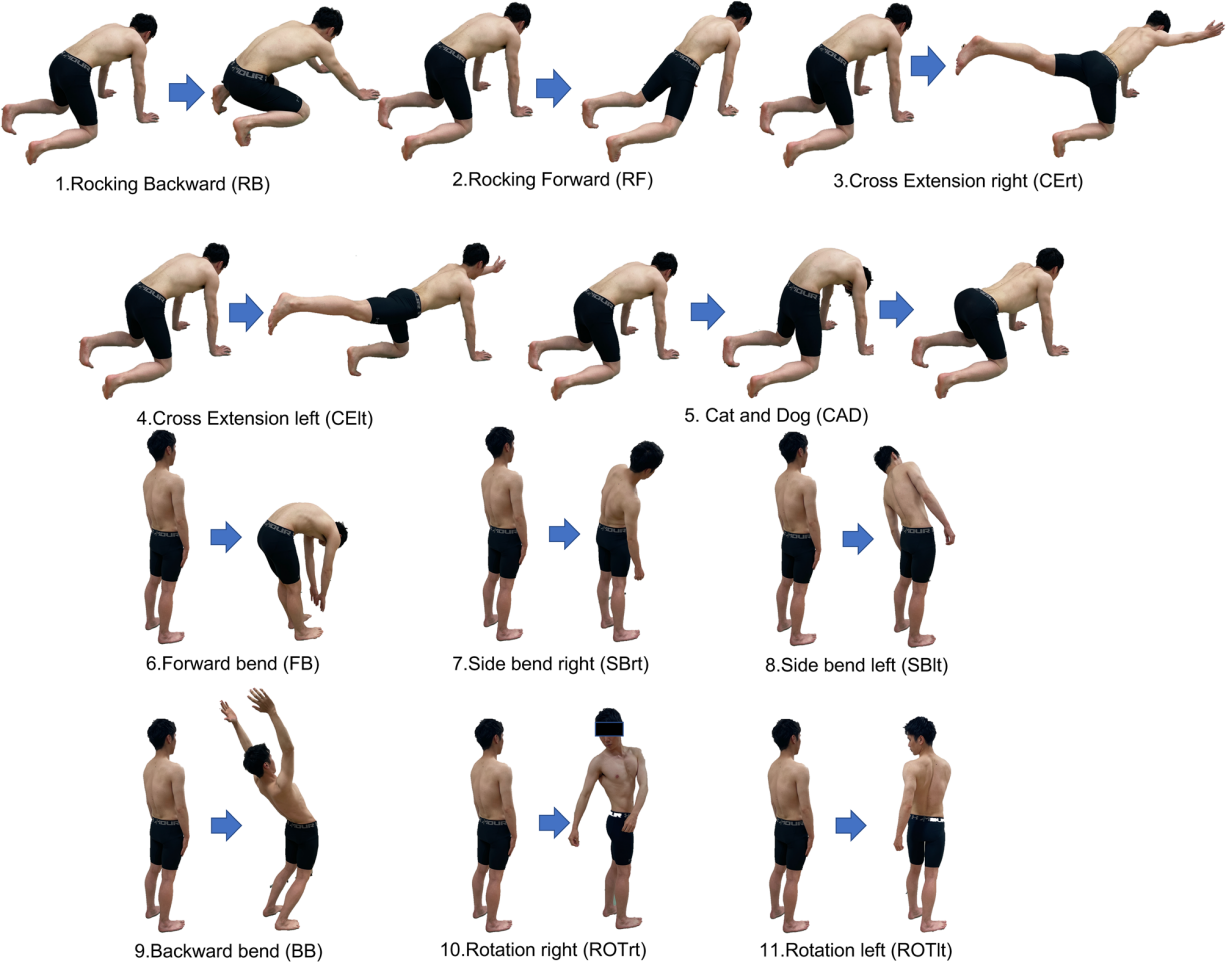
Eleven trunk movement and stability motor tasks. (1) Rocking backward (RB), (2) rocking forward (RF), (3) cross extension right (Cert), (4) cross extension left (Celt), (5) cat and dog (CAD), (6) forward bend (FB), (7) side bend right (SBrt), (8) side bend left (SBlt), (9) backward bend (BB), (10) rotation right (ROTrt), (11) rotation left (ROTlt).

Sixteen healthy volunteers (aged 21–35 years; 8 males and 8 females) participated in the study. All of them participated in the first session, and 13 additionally participated in the second session approximately one week after the first session for assessment of the variability of trunk muscle synergies between sessions. Each participant provided written informed consent for participation in the study. The study was conducted following the principles of the Declaration of Helsinki and approved by the local ethics committee of the University of Tokyo (746). During both sessions, the participants were asked to perform the 11 trunk-related movement and stability tasks (Fig. 1). A detailed description of the tasks is presented in Supplementary Information. Each task was repeated eight times, and the order of the tasks was randomly assigned.

### Data collection

Bilateral surface EMG was performed for six spinal and abdominal muscle groups: rectus abdominis (RA) (3 cm lateral to umbilicus)^36^, oblique externus (OE) (15 cm lateral to umbilicus)^37^, erector spinae at L3 (ESL3) (3 cm lateral to the L3 spinous process)^36^, erector spinae at Th9 (EST9) (5 cm lateral to the T9 spinous process)^38^, erector spinae at Th1 (EST1) (5 cm lateral to the T1 spinous process)^38^, and latissimus dorsi (LD) (lateral to T9 over the muscle belly)^37^. The activities of the muscles were recorded using a wireless EMG system (Trigno Wireless System, DELSYS, Boston, MA, USA). Each electrode had an inter-electrode spacing of 10 mm. The EMG signals were bandpass filtered (20–450 Hz), amplified (with a 300-gain preamplifier), and sampled at 1000 Hz. To prepare the skin for electrode placement, natural oil and other contaminants were removed from the skin surface with an alcohol swab. The starting point was initiated with the verbal cue “go” when the examiner manually pressed the electrical trigger once^10,39^. After participants completed the tasks and returned to a resting posture for approximately 1 s, the examiner manually pressed the electrical trigger twice with the verbal cue “end” to define the end of the movement^10,39^.

### EMG processing

Raw analog EMG signals were high-pass filtered at 30 Hz to remove motion artifacts and demeaned. The signals were full-wave-rectified and low-pass filtered at 10 Hz, using a fourth-order Butterworth filter. The smoothed EMG envelopes were time-interpolated to generate 200 timepoints between the start and end points for each trial.

Similar to previous studies^10,22,39–41^, we created concatenated EMG matrices from all 11 trunk motor tasks to obtain an “all-task” EMG matrix for each participant (that is, the matrix was composed of 12 muscles × no. of repetitions (8) × 200 samples of the 11 single-task EMG matrices) to extract trunk muscle synergies across all tasks. The EMG signal from each muscle was normalized to the maximum amplitude across the tasks. Each muscle vector in the data matrix was standardized to have unit variance to ensure that the activity in each muscle was equally weighted.

### Muscle synergy analysis

We applied NMF to the all-task EMG matrix to extract muscle synergies. NMF has previously been described as a linear decomposition technique^6,9^ according to Eq.  (1):

1 $$M=W\cdot C+e$$

where M (m × t matrix, m is the number of muscles, t is the number of time samples, M_ij_ represents the amplitude level of EMG of muscle i at timing j) is a linear combination of muscle synergies, *W* (*m* × *n* matrix, n is the number of muscle synergies), *C* (*n* × *t* matrix, representing temporal patterns), and *e* is the residual error matrix. NMF was applied to extract each possible *n* value from 1 to 12 from each dataset. The variance accounted for VAF by the reconstructed EMG (*M*) was calculated at each iteration to extract the optimal number of muscle synergies^42^. VAF was defined as the 100 × square of the uncentered Pearson’s correlation coefficient^42,43^. Because NMF utilizes the random initialization of W and C, the algorithm may have been stuck in a suboptimal local minimum. Thus, each synergy extraction was repeated 50 times and the iteration with the highest VAF was maintained^10,39,44,45^. In this study, we extracted trunk muscle synergies from all-task EMG matrices. We utilized the criterion, VAF > 0.9, to identify the optimal number of synergies commonly used in the literature^10,26,39,46–48^. It was suggested that the criterion, VAF > 0.9, ensures a sufficient representation of the data^49^, although this is still debated^50,51^. Using the criteria, we extracted trunk-related muscle synergies with the mean number 5.13 ± 0.83 [mean ± standard deviations (SD)]. To facilitate the comparison of participants and assess the inter-session variability of trunk muscle synergies, we used the same number of muscle synergies as the rounded mean number of synergies across participants, that is, five extracted from all-task EMG matrices for further analyses^44,52^.

**Figure 2:**
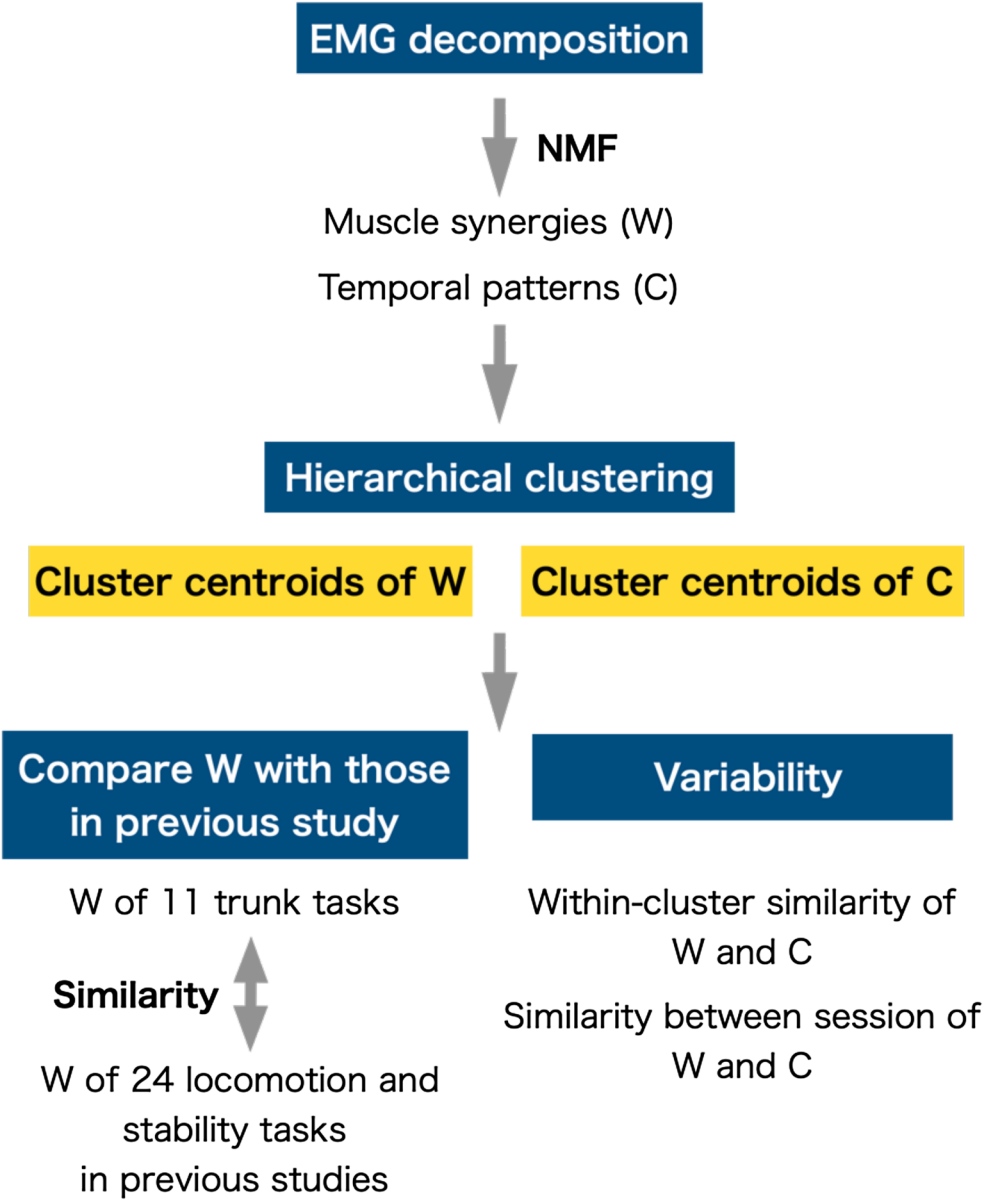
Flow-chart of the analysis trunk muscle synergies (W) and their temporal patterns (C). W and C were extracted using the NMF algorithm. To determine if the extracted synergies in the current study represented the same underlying a variety of locomotion and stability tasks found in our previous study^10^, the similarities found using the scalar product of the cluster centroids of W in the current study, and those extracted in the previous study (excluding muscle weighting components in the lower limbs) were computed. The analysis of variability was focused on within-cluster similarities and similarities between session in both W and C. The similarity values for W and C were computed using scalar product and the r_max_ coefficient using the cross-correlation function, respectively.

Figure 2 presents the flow-chart for the analysis trunk muscle synergies and their temporal patterns. First, we sorted the extracted muscle synergies into five groups using a hierarchical cluster analysis in MATLAB (“linkage” function, MathWorks, Inc., Natick, MA, USA)^22^. To determine if the five extracted muscle synergies during the 11 selected trunk motor tasks were similar to those during the 24 locomotion and stability tasks in the trunk and lower limbs in our previous study^10^, we calculated the similarity using the scalar product of the cluster centroids of muscle synergies in the current study and those extracted in our previous studies, excluding muscle weighting components in the lower limbs. Regarding the variability of trunk muscle synergies and their temporal patterns between participants, we computed the similarity by averaging the values of all pairwise dot products between muscle synergies and the r_max_ coefficient using the cross-correlation function between temporal patterns in each cluster as the variability between participants (the within-cluster similarity)^22,48^. For the variability between sessions, we sorted five trunk muscle synergies for each participant during the first and second sessions based on the centroids of the muscle synergies calculated from the analysis of the variability between participants^53^. The scalar product was computed for every pair of muscle synergies between the centroids and muscle synergies for each participant. Then, we selected the pair with the highest value of similarity, and the muscle synergies involved in that pair were removed from the set. The highest similarity among the remaining sets was chosen, and the pair was removed until all muscle synergies were matched with their best matching pair. This resulted in similar synergies, and the corresponding temporal patterns were of the same order across all participants and sessions. We computed the similarities of the reordered muscle synergies and the corresponding temporal patterns of the sessions for each participant (similarity between sessions).

### Statistics

We compared the intra-cluster similarities of the five clusters, the similarities of the sessions for muscle synergies, and the corresponding temporal pattern. The values were compared using the Kruskal–Wallis test, which is a non-parametric method for multiple comparisons of independent samples^54^, as a normal distribution was not observed in the data (tested using the Shapiro–Wilk test). When the Kruskal–Wallis test showed significant effects, multiple comparison post-hoc analyses were performed using the Mann–Whitney *U* test with Bonferroni adjustments. The significance level for all tests was set at *p* < 0.05.

## Results

### EMG patterns of all 11-trunk stability and movement tasks

**Figure 3:**
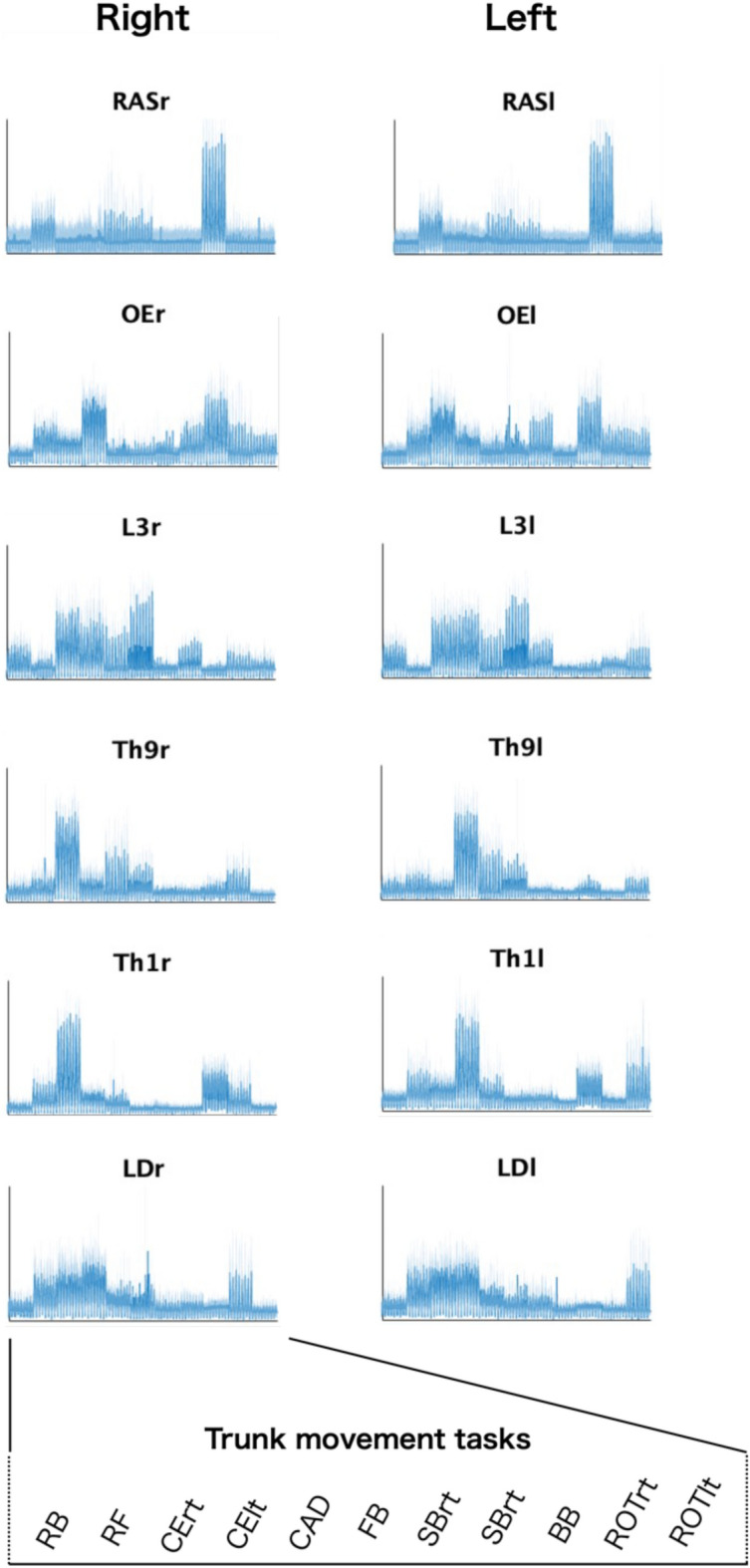
Muscle activation patterns of 1 l trunk movement tasks. The concatenated EMG envelope of 1 l trunk stability and movement tasks is shown. The mean of the EMG envelopes for 16 participants is plotted as a line and the standard deviation as a shading around it. The amplitude is normalized to the maximum value for each muscle over all tasks and standardized to have a unit variance to equally weight the EMG activity across all muscles before each synergy extraction procedure.

Figure 3 presents the concatenated EMG envelopes of all 11 tasks for all the participants. Briefly, the RAS, OE, and L3 LD presented symmetrical patterns between the right and left sides. In contrast, Th9 and Th1 cells showed asymmetrical patterns.

## Trunk muscles synergies underlying 11 trunk stability and movement tasks

**Figure 4:**
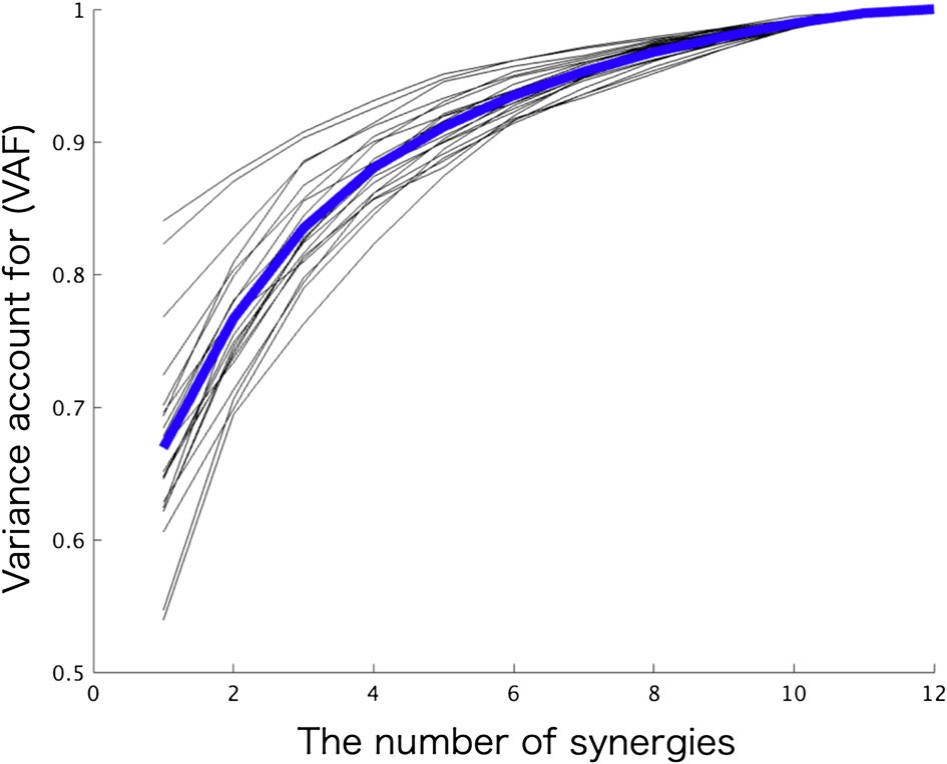
Individual (black thin lines) and participant (blue thick line) means of the percentage of variability accounted for (VAF). When 5 synergies were extracted, the mean value of VAF is 91.3% (± 0.02).

**Figure 5:**
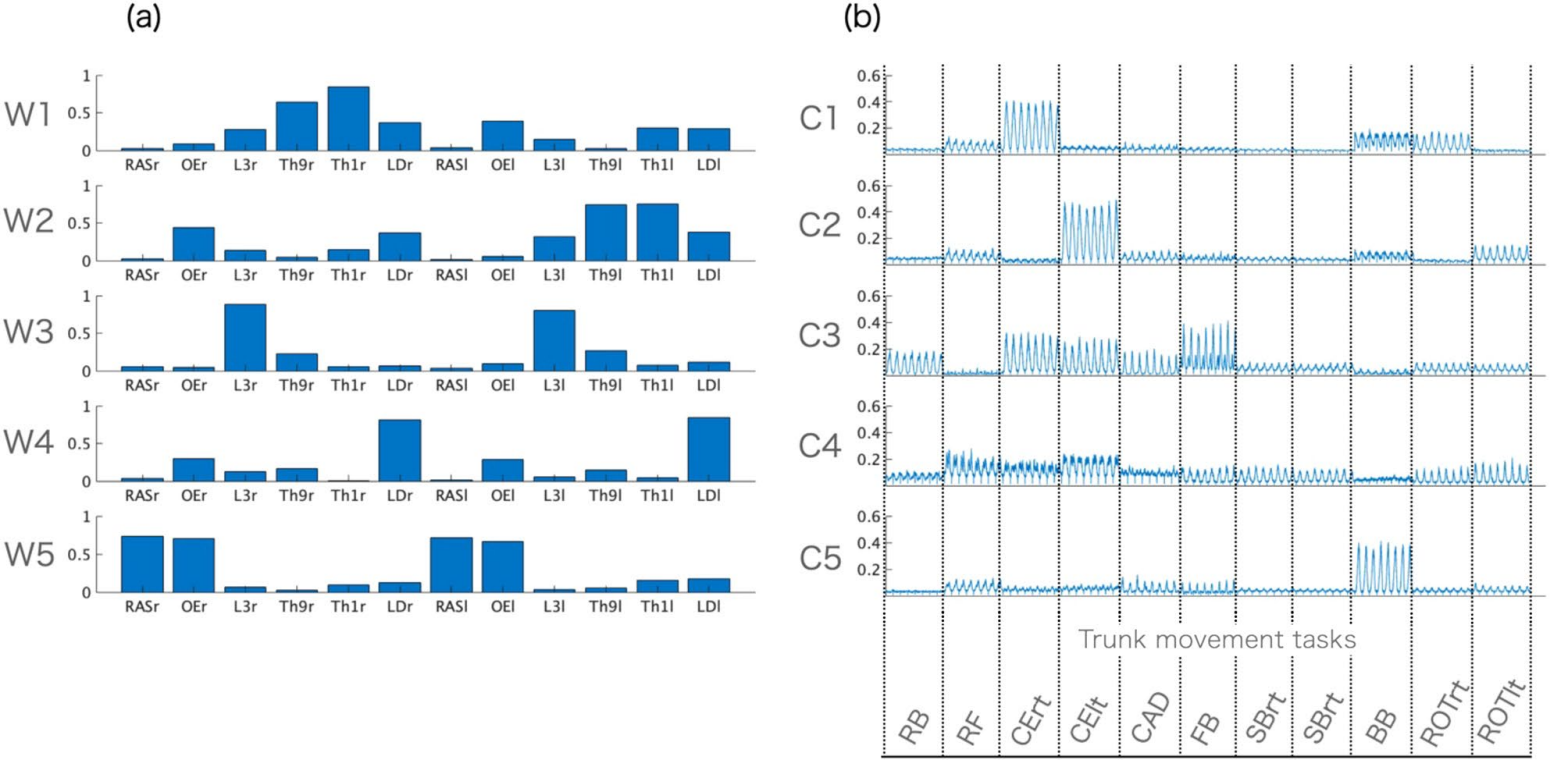
Representative (**a**) five-trunk muscle synergies (W1–W5) and (**b**) their temporal patterns (C1–C5). Non-negative matrix factorization on the concatenated electromyography (EMG) envelopes of 11 trunk motor tasks extracted five trunk muscle synergies for each participant. We sorted the trunk muscle synergies of all participants into five groups using hierarchical cluster analysis.

Figure 4 shows the VAF for each number of muscle synergies. When five synergies were extracted, the mean value of the VAF was 91.3% (± 0.02). Figure 5 shows the muscle synergy (W) cluster centroids (five groups based on muscle synergies using hierarchical cluster analysis) and their corresponding temporal patterns (C). W1 mainly represents the activation of the right Th1, Th9, and LD muscles, with the activation of the left OE. The similarity between W1 and the corresponding muscle synergy in a previous study^10^ was 0.84. C1 shows that W1 is largely activated during the cross-extension right. W2 represents the asymmetric pattern of W1; it shows the activation of the left Th1, Th9, and LD muscles with the activation of the right OE. The similarity between W2 and the corresponding muscle synergy in a previous study^10^ was 0.84. The corresponding C2 shows that W2 mainly activates the cross-extension left.

W3 mainly worked with the bilateral activation of the L3 muscles. The similarity between W3 and the corresponding muscle synergy in a previous study^10^ was 0.98. The corresponding C3 shows that W3 activates during the rocking backward, the cross extension of both sides, and the forward bend tasks. Similarly, W4 mainly represents the bilateral LD muscles. The similarity between W4 and the corresponding muscle synergy in a previous study^10^ was 0.60. The corresponding C4 shows that W4 is activated during the rocking forward and the cross extension of both sides. However, it showed relatively low activation levels relative to the other temporal pattern components. Lastly, W5 mainly involves bilateral activation of the RAS and OE muscles. The similarity between W5 and the corresponding muscle synergy in a previous study^10^ was 0.99. They worked during the backward bend task, as shown by C5.

### Intra-cluster and inter-session similarities of muscle synergy and temporal activation

Table 1 summarizes the results for the intra-cluster and inter-session similarities in W and C. Overall, the medians and ranges of these similarities varied. For the intra-cluster similarity, the median values were 0.73 to 0.86 across W and 0.64 to 0.75 for C. The lowest and highest values of the intra-cluster similarity were 0.17 and 0.99 across W and 0.42 and 0.90 across C. For the inter-session similarities, the median values were 0.81 to 0.96 across W and 0.74 to 0.84 across C. The lowest and highest values of similarity between sessions were 0.06 and 0.99 for W and 0.49 and 0.91 for C.

**Table 1:** Summary of the results for the intra-cluster similarity and inter-session similarity of muscle synergy (W) and temporal activation (C).

	W1	W2	W3	W4	W5
Within cluster similarity	0.79 (0.27–0.97)	0.73 (0.17–0.92)	0.86 (0.33–0.98)	0.86 (0.44–0.97)	0.84 (0.20–0.99)
Similarity between sessions	0.88 (0.40–0.98)	0.81 (0.27–0.99)	0.92 (0.06–0.98)	0.89 (0.06–0.98)	0.96 (0.64–0.99)

	C1	C2	C3	C4	C5
Within cluster similarity	0.74 (0.42–0.90)	0.75 (0.51–0.86)	0.65 (0.52–0.84)	0.67 (0.48–0.81)	0.64 (0.44–0.85)
Similarity between sessions	0.84 (0.78–0.91)	0.82 (0.57–0.89)	0.74 (0.49–0.86)	0.77 (0.53–0.86)	0.77 (0.65–0.91)

Each value is provided as a mean and the ranges.

**Figure 6:**
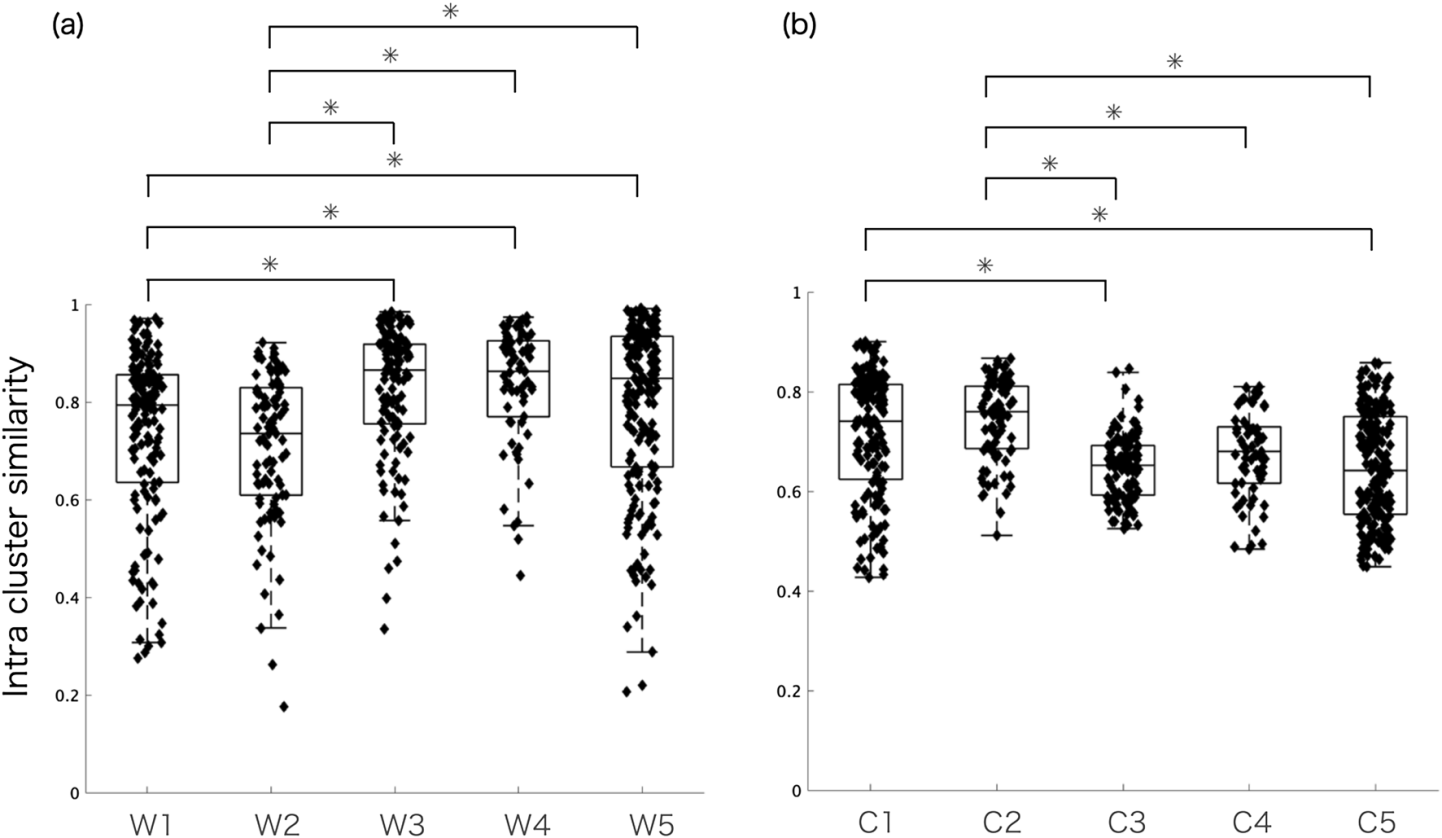
Intra-cluster similarity of (**a**) muscle synergies (W1–W5) and (**b**) temporal patterns (C2–C5). Kruskal–Wallis test showed p < 0.05 for both W and C. Multiple comparisons show statistically significant differences between W and C as follows: W1 and W3, W1 and W4, W1 and W5, W2 and W3, W2 and W4, and W2 and W5 (p < 0.05). C1 and C3, C1 and C5, C2 and C3, C2 and C4, and C2 and C5 (p < 0.05).

Figure 6 shows the differences in the intra-cluster similarity in W and C. The Kruskal–Wallis test showed p < 0.05 for both W and C. Multiple comparisons of W showed significant differences between W1 and W3, W1 and W4, W1 and W5, W2 and W3, W2 and W4, and W2 and W5 (p < 0.05). Similarly, multiple comparisons of C showed significant differences between C1 and C3, C1 and C5, C2 and C3, C2 and C4, and C2 and C5 (P < 0.05).

**Figure 7:**
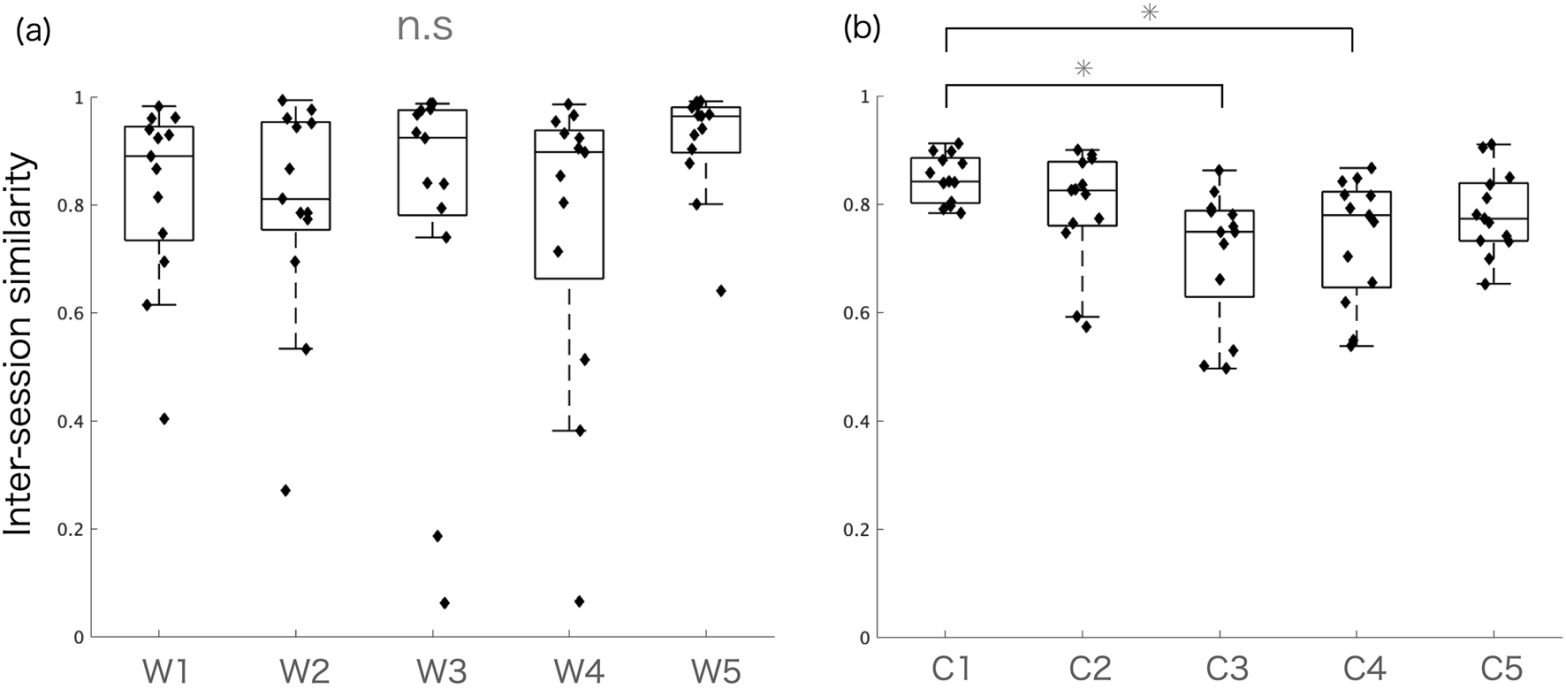
Inter-session similarity of (**a**) muscle synergies (W1–W5) and (**b**) temporal patterns (C2–C5). Kruskal–Wallis test showed p = 0.2084 for W and p < 0.05 for C. Multiple comparisons show statistically significant differences in C as follows: C1 and C3 and C1 and C4 (p < 0.05).

Figure 7 shows the differences in the similarity between sessions W and C. The Kruskal–Wallis test showed no significant differences in the similarity between W (p = 0.2084) and significant differences between C (p < 0.05). Thus, multiple comparisons of C showed significant differences between C1 and C3 and between C1 and C4 (p < 0.05).

## Discussion

In the present work, we extracted five trunk muscle synergies from 12 trunk muscle activations (six unilateral muscles) during 11 multidirectional and stability trunk-related motor tasks. Even in a highly variable task context, five trunk muscle synergies were used to reconstruct the original EMG data well (i.e., the mean value of VAF was 91.3%). Overall, while the similarities of trunk synergies between participants and sessions appeared to be comparable with those reported by some previous studies on other body parts, these metrics had a broad range, reflecting some level of redundancy of the musculoskeletal system in healthy individuals. Furthermore, the significant differences in variability between the synergies reflect the different features of trunk synergy organization and strategies for addressing various mechanical demands of a motor task.

Regarding the similarity between participants, our findings presented the median values of similarity ranging from 0.73 to 0.86 for five trunk muscle synergies (W) and from 0.64 to 0.75 for their temporal patterns (C). These inter-cluster similarities appear to be greater than those in highly variable upper-limb movements found in a previous study, although the similarity of temporal patterns was not assessed^22,55^. This suggests that trunk muscle synergies have more robust features as the trunk requires both stability and movement even in the loosely constrained task scenario, compared with the upper limbs, which primarily function as a wide range of manipulations with many degrees of freedom and involve motor cortical neurons with complex and heterogeneous motor patterns^39^. However, as shown in Table 1 and Fig. 6, the ranges of the individual pair values for both W and C were broad. That is, the lowest and highest values of similarity were 0.17 and 0.99 for W and 0.42 and 0.90 for C. Thus, it is strongly suggested that some healthy participants shared similar control strategies for highly variable scenarios of the trunk while other did not. The lower similarity values may reflect the redundancy of the musculoskeletal system in which different participants have different motor control options for executing the same motor task^23^. We further found that muscle synergies of bilateral muscle activations (i.e., W3 to W5) presented significantly higher intra-cluster similarity than those of unilateral muscle activations that involved different levels of paraspinal muscles (i.e., W1 and W2) (Fig. 6). A previous study utilized network analysis to investigate the relationship between anatomical and functional connectivity, defined by the intermuscular coherence of muscle activation^31^. They found bilateral connectivity between the abdominal and back muscles, as opposed to the upper and lower limbs that primarily connect unilaterally across different muscle groups^31^. The finding of bilateral connectivity may reflect the fact that sharing common input with bilateral trunk muscles is strongest in spinal motor neurons that innervate muscle pairs that are anatomically and functionally closely related^56^. These embedded structures may lead to a higher similarity of bilateral muscle patterns of W3, W4, and W5 between participants when compared to the unilateral patterns of W1 and W2. In contrast, an increase in the number of unilateral trunk muscle involvements in W1 and W2 with a different nerve supply may allow motor control strategies in less similar manners between participants^56^. Furthermore, we found that the less similar features of unilateral muscle synergies in W1 and W2 produced more consistent temporal patterns (C1 and C2), as shown in Fig. 6. Here, C1 and C2 showed that W1 and W2 were only activated in the right and left cross-extension tasks, in which participants were asked to stabilize the trunk in all four positions with the ipsilateral upper limb and the contralateral lower limb elevated (Fig. 5). It is possible that when executing the highly constrained trunk stability tasks, participants present more consistent activation patterns regardless of the highly variable synergy organization (i.e., W1 and W2). However, significantly lower similarities of C3, C4, and C5 than those of C1 and C2 were found. As shown in Fig. 5, C3 to C5 involved rocking backward, forward bend, and backward bend and employed a larger excursion of trunk movement. This reflects that the large trunk movement allows each participant to find many output solutions and presents highly variable recruitment patterns.

We found that the similarity between sessions was 0.81 to 0.96 across W and 0.74 to 0.84 across C, with no significant differences between W and C except between C1 and C3 and C1 and C4 (Table 1 and Fig. 7). For W, our results were comparable or may be more similar to those of a previous study that investigated the inter-session similarity of forearm muscle synergies for various daily life grip postures, which was between 0.70 and 0.85^19^. This suggests that trunk muscle synergies may be more robust between sessions than those of the upper limbs. In contrast, another study found a higher similarity (> 0.9) between sessions for lower limb muscle synergies during daily life activities, such as walking, running, and ascending and descending stairs^20^. However, they extracted lower-limb synergies and analyzed the inter-session similarity for each task independently, resulting in the relatively high mechanical constraints demanded by a task. It has also been suggested that muscle synergies related to locomotion are determined by early development and robustly preserved into adulthood^57,58^, reflecting the existence of lower-level neural control structures in the lower limbs^11,48,59^. Thus, these mechanical and neural constraints may be associated with the higher similarity between sessions in lower limb synergies compared to trunk synergies in our study. Furthermore, as shown in Table 1 and Fig. 6a, the individual values of inter-session similarity presented broad ranges. Here, the lowest and highest values of the similarity were 0.06 and 0.99 for W and 0.49 and 0.91 for C. Notably, there were few cases that showed 0.4 or lower similarity for W between sessions. In contrast, the similarity of C did not show such low cases. This suggests that although muscle synergies are dissimilar, they may share similar temporal patterns. As such, the very low similarity of muscle synergies between sessions could reflect the fact that some healthy participants may use different muscle recruitment options in a different session during the same motor tasks due to the redundancy of the musculoskeletal system^23^. This was further supported by the loosely constrained task scenario in the current study without any tempo for the execution of the task using a metronome that facilitates the same motor output patterns between sessions^21^. Furthermore, some level of variability between sessions can also be attributed to experimental protocols, including the fact that electrodes were not placed on each trunk muscle in exactly the same way between sessions, or other physical factors, such as skin conditions and fatigue^19,60^. Thus, variability due to experimental factors needs to be considered when evaluating motor control strategies in different populations in longitudinal studies with multiple sessions^55^.

Muscle synergy analysis can be a useful tool in assessing specific sensorimotor profiles to define targets for the rational development of novel motor control interventions that enhance neural plasticity and improve motor recovery^61^. Here, we analyzed the muscle synergies during 11 multidirectional trunk movements and stability motor tasks in healthy participants for clinical application. Specifically, the datasets for intra-cluster similarity and inter-session similarity were assessed to provide reference data on variability in trunk coordination patterns in healthy individuals. Thus, it will be interesting to investigate these variability indices for other populations (i.e., musculoskeletal and neurological diseases or athletes) to assess whether trunk motor control strategies present stereotypes or diverse features in the population of interest. For example, there is evidence that each participant has diverse adaptations of muscle synergies in response to experimentally induced pain during neck movements^62^, multidirectional reaching^63^ and locomotion^64^. These findings highlight subject-specific solutions with many degrees of freedom in the musculoskeletal system to avoid further tissue damage while performing motor tasks. On the other hand, athletes exhibited similar neuromuscular control strategies for complex motor skills, reflecting stable and precise execution of movements achieved through similar training experiences^27^. To the best of our knowledge, the analysis of muscle synergies during highly variable trunk motor task behaviors has not been applied in research to reveal distinct features of trunk control in the population of interest. Our methods using these muscle synergy analyses would allow the capture of a broad spectrum of movement characteristics in the trunk and provide efficient biomarkers as indicators of pathological processes and responses to therapeutic interventions based on motor control^65^.

## Supplementary Information

Supplementary Table 1.

## Data Availability

The datasets analyzed during the current study are available from the first or corresponding author on reasonable request.
