# Supplementary material for: Variability of trunk muscle synergies underlying the multidirectional movements and stability trunk motor tasks in healthy individuals
Source: Sci Rep. 2023 Jan 21;13:1193. doi: 10.1038/s41598-023-28467-6 (PMC9867711; doi:10.1038/s41598-023-28467-6)
Supplement: Supplementary file 1 — Supplementary Table 1. [file 41598_2023_28467_MOESM1_ESM.pdf]

**Supplementary Information**

**Title:** Variability of trunk muscle synergies underlying the multidirectional movements and stability trunk motor tasks in healthy individuals

Hiroki Saito[1,2], Hikaru Yokoyama[3*], Atsushi Sasaki[4, 6], Kazuya Matsushita[5], Kimitaka Nakazawa[1]

[1] Graduate School of Arts and Sciences, Department of Life Sciences, The University of Tokyo, Tokyo, Japan

[2] Department of Physical Therapy, Tokyo University of Technology, Tokyo, Japan

[3] Institute of Engineering, Tokyo University of Agriculture and Technology, Tokyo, Japan

[4] Graduate School of Engineering Science, Department of Mechanical Science and Bioengineering, Osaka University,

[5] Aobadai Takeda orthopedic, Tokyo, Japan

[6] Japan Society for the Promotion of Science, Tokyo, Japan

Correspondence to: Hikaru Yokoyama

Institute of Engineering, Tokyo University of Agriculture and Technology, Tokyo, Japan

Email: h-yokoyama@go.tuat.ac.jp

**Supplementary Table 1. Full descriptions of 11 trunk movement and stability motor tasks.**

| Task numbers | Tasks | Position | Conditions |
|---|---|---|---|
| 1 | Rocking Backward (RB) | Quadruped | A transfer of the buttock backwards ("rocking") keeping low back in neutral until knees reach approximately 130 degrees of flexion and a return. |
| 2 | Rocking Forward (RF) | Quadruped | A transfer of the buttock forward ("rocking") keeping low back in neutral until knees reach 0 degree of hip extension and a return. |
| 3 | Cross Extension right (Cert) | Quadruped | A raise of a right arm and a left leg straight out and a return. |
| 4 | Cross Extension left (Celt) | Quadruped | A raise of a left arm and a right leg straight out and a return. |
| 5 | Cat and Dog (CAD) | Quadruped | A round of back and drop a chin to a chest (cat) and a lift of head up and an arch of back down toward a floor (dog) and a return. |
| 6 | Forward bend (FB) | Standing | A bend of trunk forward as far as possible and a return. |

| | | | |
|---|---|---|---|
| 7 | Side bend right<br>(SBrt) | Standing | A bend of trunk to the right side as far as possible and a return. |
| 8 | Side bend left<br>(SBlt) | Standing | A bend of trunk to the left side as far as possible and a return. |
| 9 | Backward bend<br>(BB) | Standing | A bend of trunk backward as far as possible and a return with arms raised. |
| 10 | Rotation right<br>(ROTrt) | Standing | A rotation of trunk to the right side as far as possible and a return. |
| 11 | Rotation left<br>(ROTlt) | Standing | A rotation of trunk to the left side as far as possible and a return. |
